# Perceptions of mothers with preterm infants about early communication development: A scoping review

**DOI:** 10.4102/sajcd.v67i1.640

**Published:** 2020-01-29

**Authors:** Elanie van Schalkwyk, Samantha Gay, Julia Miller, Elani Matthee, Berna Gerber

**Affiliations:** 1 Division of Speech-Language and Hearing Therapy, Faculty of Medicine and Health Sciences, Stellenbosch University, Cape Town, South Africa

**Keywords:** mother, perception, preterm infant, early communication development, communication, interaction

## Abstract

**Background:**

Preterm infants are at risk of communication disorders or delays, and their mothers experience various difficult emotions and realities. These communication difficulties could be effectively prevented or addressed through the provision of appropriate maternal support. Maternal perceptions regarding early communication–interaction and development in preterm population should thus be well understood by health professionals. Previous studies have focussed on parents’ and patients’ perceptions of medical information received from health professionals. Limited research, however, has been undertaken on maternal perceptions of early communication development in preterm infants, specifically in the South African context.

**Objectives:**

The study aims to summarise the range and the nature of available research in the fields of early communication development and intervention in preterm infants, specifically maternal perceptions thereof.

**Method:**

A scoping review methodology comprising five phases was used. Data were extracted from the final selection of 12 articles and analysed through quantitative and thematic techniques.

**Results:**

The results of the scoping review indicate that the defined research field is in a developing phase. Mothers mainly experience negative emotions and have limited knowledge regarding communication interaction with preterm infants. Furthermore, hospitalisation has been experienced as a barrier to natural communication–interaction between mother and infant.

**Conclusion:**

Based on these results, it is recommended that primary research be conducted with the mothers of preterm infants to establish the most effective strategies for communication–interaction training with this vulnerable population. A further recommendation would be to increase awareness of early communication development and intervention in the preterm population amongst both parents and health professionals.

## Introduction

An estimated 15 million babies are born prematurely every year (World Health Organization [WHO], 2018). Preterm birth is defined as the birth before 37 weeks of pregnancy (WHO, 2018). Approximately 84,000 of these births occur in South Africa, and 10% of the preterm infants born in South Africa do not survive in spite of the fact that the majority of preterm births occurs at healthcare facilities (WHO, 2018). Many caregivers in developing countries are exposed to a myriad of factors that may increase the risk of preterm birth and challenge caretaking following preterm birth (WHO, 2018). The majority of South Africans live in low socioeconomic conditions (Statistics South Africa, 2017), and this could be viewed as a serious threat to childhood outcomes (Dawes, Biersteker, & Irvine, 2008). Poor living environments, dangerous working environments and limited social networks and support for mothers are common amongst populations with low socioeconomic status. These factors increase the risk of preterm births and complicate the task of taking care of preterm infants.

The mothers of preterm infants have a different communication–interaction experience with their infants than the mothers of full-term infants. Preterm infants are born whilst they are still neurologically, physiologically and anatomically unprepared for life outside the mother’s womb (Crisp, 2006). Therefore, they are less alert and responsive than full-term infants (Pascoe, Bissessur, & Mayers, 2016) and display limited interaction with their environment. Communication development unfolds in the context of an infant’s social environment. The nature of mother–infant interactions plays a critical role in supporting and shaping communication development. The factors that influence the quality of mother–child interactions must thus be well understood to provide effective early communication intervention for preterm infants (Loi et al., 2017).

Feeding skills of preterm infants are also often compromised, thereby hindering breastfeeding and bonding between the mother and infant dyad (Flacking, Ewald, Nyqvist, & Starrin, 2006). Preterm infants generally require intervention from a team of health professionals and may undergo frequent and/or prolonged hospitalisation. Hospitalisation includes in-patient services such as admission to Neonatal Intensive Care Unit (NICU) and other relevant wards, as well as out-patient services such as high-risk follow-up clinics and other relevant therapeutic interventions. The hospital setting, specifically the NICU, is a novel environment for most mothers and is often experienced as challenging or intimidating (Swift & Scholten, 2010). A broad range of personal and contextual factors (WHO, 2018) therefore contributes to inhibit prolonged periods of communication–interaction between the mother and infant dyad. This often leads to the formation of negative maternal perceptions regarding their preterm infant’s, as well as their own capabilities.

A mother’s negative perceptions regarding her preterm infant and parenting abilities may inhibit the development of normal communication–interaction. Rapid communication development takes place during the first 2 years of infant’s life (Beuker, Rommelse, Donders, & Buitelaar, 2013). It is during this period that crucial skills and opportunities – such as joint attention and engagement with the environment – are developed and provided to lay a sound foundation for the social, emotional and academic development of the child (Crisp, 2006). Studies have shown that infants who experience positive interactions with their mothers display improved cognitive and linguistic development (Nicolaou, Rosewell, Marlow, & Glazebrook, 2009). Negative maternal perceptions often lead to reduction in interaction with the preterm infant and thus limit the preterm infant’s experience of successful communication.

Contact between the mother and the healthcare professional during in-patient (e.g. NICU admissions) or out-patient (e.g. high-risk follow-up clinics) services provide a window of opportunity to support mother–infant interaction and bonding, thereby preventing or mitigating negative maternal perceptions. Early identification of communication disorders/delays in preterm infants is critical to ensure optimal communication development, as this provides us with an opportunity to implement both preventative and therapeutic intervention services (Rossetti, 2001). Preventative intervention includes health promotion programmes focussed on mothers and infants such as the *Mom. Connect* or *First 1000 Days* initiatives of the National Department of Health (2018) and Western Cape Government (2018).

The rationale for this scoping review was that an enhanced understanding of existing evidence regarding maternal perceptions may lead to improved early intervention service delivery to the mother–infant pair. This creates the potential to improve interaction–attachment and bonding, and thus communication outcomes, for both mother and the preterm infant. The following research question was therefore articulated: *What is the nature of the research literature on the perceptions of mothers of preterm infants regarding the early communication development of their babies*? The aims of our study were twofold. Firstly, to map the literature in terms of time (year of publication), setting (location and context in which it was created) and types of research methodology employed. Secondly, to describe this body of research in terms of its results that pertain to maternal perceptions regarding early communication development and intervention in preterm infants. This study therefore aims to serve as a foundation for further primary research and recommendations for clinical practice with the goal of improving early intervention service delivery to preterm infants and their mothers.

## Methodology

**FIGURE 1:**
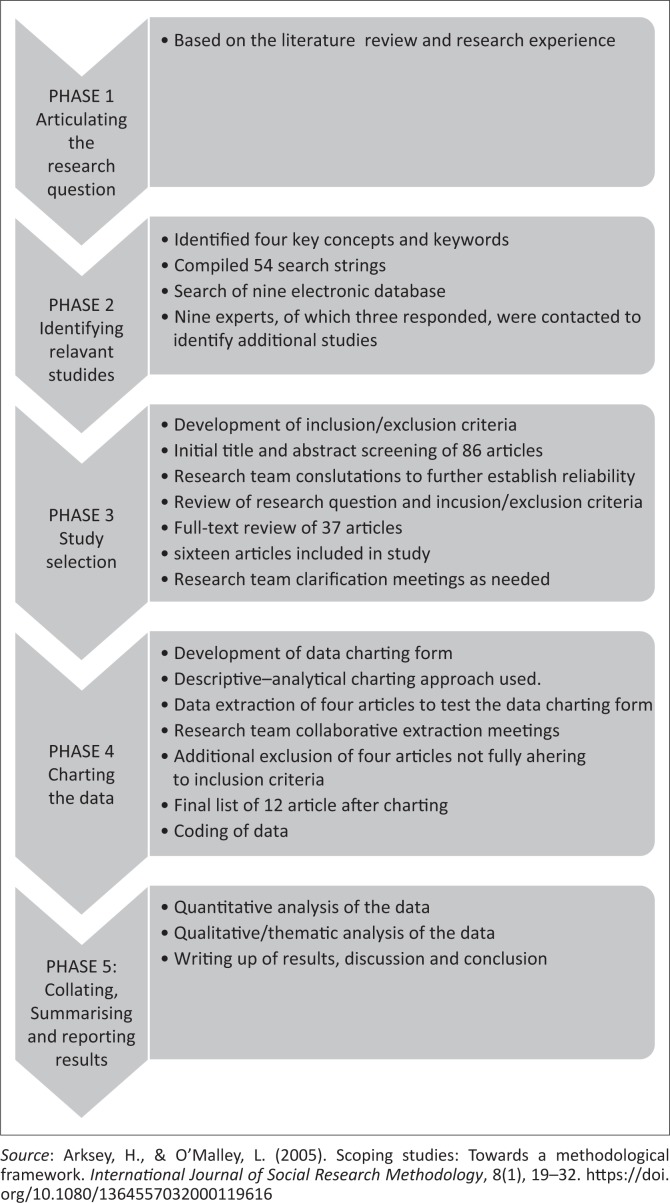
Summary of methodology.

The scoping review method is defined as a form of knowledge synthesis that addresses an exploratory research question. Key concepts, types of evidence and gaps in a defined research field are mapped through systematically searching, selecting and synthesising existing knowledge (Colquhoun et al., 2014). This scoping review comprised five phases as outlined by Arksey and O’Malley (2005) and summarised in Figure 1.

The four key concepts that are to be addressed in a relevant manner for inclusion of a research paper in the scoping study are listed in Table 1. These concepts and related terminologies were identified during a literature search within the defined research field.

**TABLE 1:** Summary of key concepts and related terminology.

Key concept	Related term	Related term
Perception(s)	Knowledge	Understanding
Mother(s)	Caregiver(s)	Parent(s)
Preterm infant(s)	Premature infant(s)	Low birth weight
Early communication development	Early development	Prelinguistic development

The inclusion criteria were established after considering the research question and the aims of the study, as described in the Introduction as well as the definitions of key concepts. Table 2 provides the inclusion criteria and the accompanying motivations (Levac, Colquhoun, & O’Brien, 2010).

**TABLE 2:** Inclusion criteria.

Inclusion criterion	Rationale
Studies that are published in English	Ensure access to the information
Studies that are fully and freely accessible to be downloaded through the Stellenbosch University Library services	Ensure access to the information obtained, and reduce financial obligations
Studies addressing four key concepts in a manner relevant to the research question and the aim of the study	Ensure relevance and thus the validity of the obtained information

**FIGURE 2:**
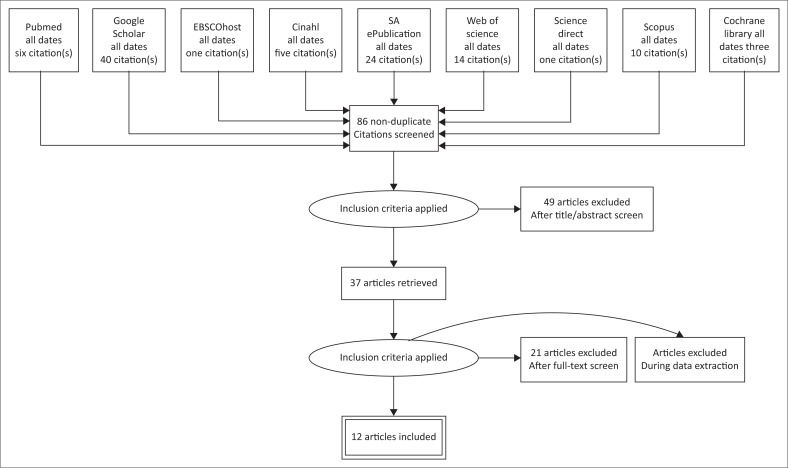
PRISMA diagram representing the number of articles found in each database.

The PRISMA diagram (Figure 2) provides the nine databases that were searched as well as the number of articles found in each database. The most recent search was performed on 12 February 2018, and studies from all dates were included as a result of the limited number of research in the defined field.

An example of the search string used to search the database Cinahl is as follows:

… (knowledge OR perception OR understanding) AND (*mother OR caregiver) AND (preterm infants OR premature infants OR low birth weight) AND (early communication development OR prelinguistic OR early development)

The first four researchers of this article reviewed the titles, abstracts, followed by the full text of articles identified to increase consistency (Tricco et al., 2018). All disagreements were resolved through discussion until a consensus was reached. The initial search yielded 104 articles, of which 18 articles were eliminated because of duplication. Twenty-one articles were excluded during the full-text review because they did not adhere to the four key concepts and the key words in a relevant manner. During the data extraction phase, another four articles were excluded. After charting the data from these articles, the researchers reached a consensus that the information did not sufficiently address the research question. Therefore, 12 articles were included in this scoping review, as displayed in Appendix 1.

This study followed a descriptive–analytical charting approach, which involved applying an identical analytical framework to all 12 articles included in the scoping review and collecting standard information from each article. The extraction of data involved a technique called charting, known as synthesising and interpreting qualitative data by sifting through and sorting material according to key concepts and themes (Arksey & O’Malley, 2005). Applying a consistent approach allowed the researchers to make comparisons and identify contradictions, gaps and ‘new frontiers’ amongst the included studies. The extracted data were entered into a Microsoft Excel spreadsheet. Appendix 2 demonstrates the information extracted from each article.

The goal of the final phase of this scoping review was to present an overview of all the material reviewed to answer the outlined research question, namely: ‘what is the nature of the research literature on the perceptions of mothers of preterm infants regarding the early communication development of their babies?’ In keeping with the scoping review methodology (Arksey & O’Malley, 2005), this was done without assessing the quality of the reviewed evidence or determining whether this evidence could be generalised. The year of publication, study setting (location and context) and type of study were analysed numerically to summarise the extent and nature of studies included in this review (Arksey & O’Malley, 2005) and to fulfil the first aim, namely: ‘to map the literature in terms of time (year of publication), setting (location and context in which it was created) and types of research methodology employed’. This method of analysis allowed the researchers to obtain an overview of the general information of the selected studies as well as to identify the most frequently occurring study types, settings and procedures of data collection and analysis. In order to fulfil the second aim, namely: ‘o describe this body of research in terms of its results that pertains to maternal perceptions regarding early communication development and intervention in preterm infants’, the results of the studies were analysed using content-based analysis to provide an overview of the ideas that emerged from the results of the reviewed literature and the frequency of these ideas amongst the analysed articles. Through this method, we aimed to retain clarity of the reporting strategy so that potential bias in reporting or recommendations could be determined by the reader (Arksey & O’Malley, 2005).

## Review findings

### Numerical analysis results

The year of study of publications ranged from 2002 to 2016, with an even spread of approximately one publication per year across the period.

**FIGURE 3:**
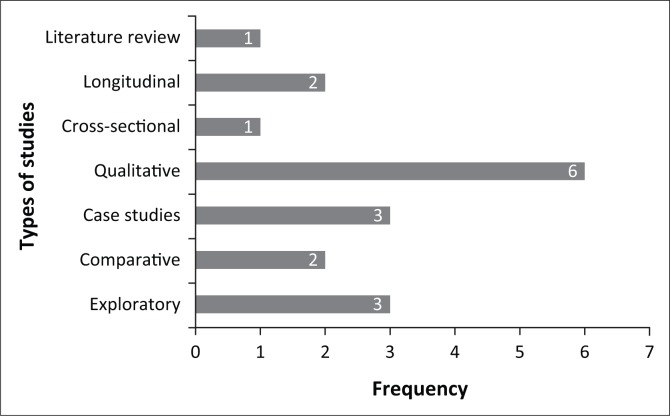
Bar graph representing the types of studies included in the scoping review.

Figure 3 illustrates the types of studies as well as the frequency of each study type amongst the set of reviewed studies.

It is important to note that some listed studies used more than one descriptor to describe their research approach. Half of the studies (i.e. six) included in the scoping review were qualitative studies. This type of research is well suited to the research topic, as the sensitive nature of the topic lends itself to smaller number of participants with results that cannot and should not be generalised too widely. Within our defined research field, only one review article was available in the form of a ‘generic’ literature review (Grant & Booth, 2009, p. 94).

The majority of studies (i.e. seven) were conducted in South Africa and in particular within NICU settings in public hospitals. The local research community should thus be commended for being relatively active in this research field. It is important to note that the context and culture of research participants greatly affects their perceptions and knowledge, and therefore the results of this scoping review should not be taken to represent the perspectives of all mothers globally. The high number of South African studies may be ascribed to the large number of risk factors for premature birth found in South Africa. These risk factors include high levels of poverty, limited formal maternal education and maternal HIV and/or AIDS (Mcinroy & Kritzinger, 2005). This finding supports our claim that speech–language pathologists, and specifically those working in the South African context, should aim to increase their knowledge and skill in delivery of service to neonatal population.

### Thematic analysis results

**FIGURE 4:**
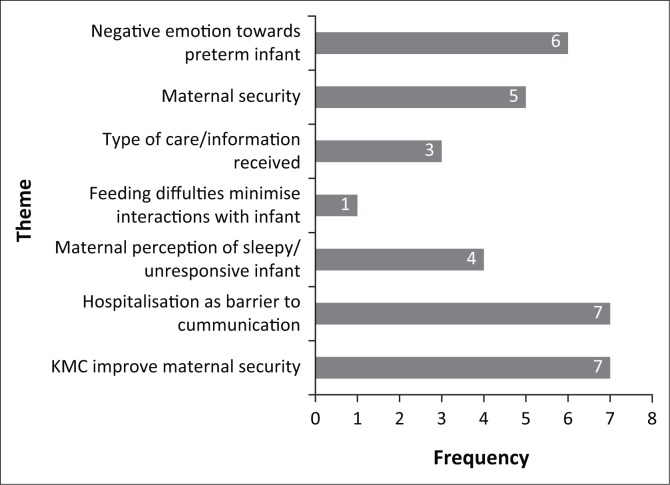
Bar graph representing the codes of relevant results from the data. KMC, Kangaroo mother care.

Seven themes emerged from the results of the included studies (the frequency of each theme is illustrated in Figure 4). These themes highlight the existing maternal perceptions regarding communication with their preterm infants. Factors that mothers perceived as barriers to communication with their preterm infants constitute the majority of the identified themes.

*Mothers experience negative emotions, such as guilt, anxiety and uncertainty, relating to their preterm infant* (Flacking et al., 2006; Leonard & Mayers, 2008; McInroy & Kritzinger, 2005; Nicolaou et al., 2009; Swift & Scholten, 2010; Tanner, 2012): These emotions could be assigned to a variety of factors such as maternal insecurity, limited communicative and feeding capabilities of infant as well as the child’s physical appearance (Crisp, 2006).

*Maternal insecurity* (Flacking et al., 2006; Kritzinger & Louw, 2003; Leonard & Mayers, 2008; Nicolaou et al., 2009; Swift & Scholten, 2010): A significant number of reviewed literature identified that mothers lacked confidence in their ability to take care of a medically compromised infant. This, ironically, pertained more to communication–interaction and independently performing daily caretaking routines, such as bathing or dressing, than complicated medical procedures or monitoring (Flacking et al., 2006). A possible reason that was suggested for this phenomenon was that information received from healthcare professionals focussed almost exclusively on medical aspects, with little attention on how to interact with and take care of a preterm infant (Swift & Scholten, 2010).

*Type of care and/or information received* (Crisp, 2006; Nicolaou et al., 2009; Swift & Scholten, 2010): The medical and physical needs of infants are often prioritised above communication–interaction when information or care is provided to mothers.

*Feeding difficulties lead to minimised interaction with infants* (Swift & Scholten, 2010): Feeding difficulties experienced by preterm infants, such as problems with breastfeeding, lead to compromised bonding between mothers and infants as well as maternal feelings of guilt. The task of feeding is complicated owing to the reduced feeding ability of infant as well as increased maternal anxiety and shifting of focus from pleasurable interaction to task completion (Flacking et al., 2006).

*Maternal perceptions of a sleepy or unresponsive infant* (Kritzinger & Louw, 2003; Nicolaou et al., 2009; Pascoe et al., 2016; Tanner, 2012): These reported perceptions lead to a feeling of one-sided interaction and uncertainty on the mother’s part (Pascoe et al., 2016).

*Hospitalisation poses a barrier to natural communication–interaction* (Crisp, 2006; Flacking et al., 2006; Kritzinger & Louw, 2003; Leonard & Mayers, 2008; McInroy & Kritzinger, 2005; Nicolaou et al., 2009; Swift & Scholten, 2010): The NICU is a novel environment for most mothers, leading to a unique and challenging experience (Swift & Scholten, 2010). Incubators, feeding tubes, respiration tubes and a variety of monitors attached to small preterm infants create both physical and emotional barriers. Strict rules and policies regarding physical handling, visiting hours and feeding schedules further limit the naturalness of interaction. The constant presence of healthcare professionals, such as nurses and doctors, are often seen as an invasion of privacy between the infant and the mother and their opportunity to freely interact with each other. This leads to the maternal perception of being redundant and a passive onlooker of the process. The attitude of healthcare professionals, especially the nurses who perform daily caretaking routines normally performed by infant’s mother, is perceived as an enabler of or barrier to interaction. Nurses who actively include the mother in this process stimulate bonding and interaction between the mother and infant dyad. Nurses who perform these routines independently create maternal perceptions of incompetency and hindered bonding and interaction.

Culture and language differences between mothers and healthcare professionals are perceived as further potential barriers to communication–interaction between mothers and their infants in hospital settings. This could be ascribed to communication breakdown occurring when health professionals attempt to explain concepts related to facilitating communication–interaction in the presence of language differences. Thus, mothers may not understand health professionals’ advice on how to facilitate the communication–interaction with their infant and may also be reluctant to implement behaviours that deviate from their cultural beliefs and practices. This perception is of special relevance when viewed in light of a multilingual and multicultural country, such as South Africa, where the culture and language of mothers and the health professionals are likely to differ (Penn & Watermeyer, 2018), and the values and practices of the profession largely reflect those of Western societies (Verdon, Blake, Hopf, Pham, & Mcleod, 2016).

*Kangaroo mother care (KMC) improves maternal perceptions of their preterm babies’ early communication capabilities* (Feldman, Eidelman, Sirota, & Weller, 2002; Flacking et al., 2006; Green & Phipps, 2015; Kritzinger & Louw, 2003; Kritzinger & Van Rooyen, 2014; McInroy & Kritzinger, 2005; Pascoe et al., 2016): A significant number (i.e. seven) of the included studies indicated that mothers who practiced KMC demonstrated a strong awareness of their role in early communication and the development thereof. Interaction between mother and infant for prolonged periods increases the mother’s ability to perceive and infer the meaning behind her infant’s behavioural signals, and to respond to them promptly and appropriately. In a comparison study, Kritzinger and Van Rooyen (2014) conclude that practicing KMC led to the formation of more realistic and positive maternal perceptions regarding early communication and interaction with their preterm infant. Mothers who practise KMC thus had a better understanding of their children and respond in a more natural manner during interactions with their infants than mothers who did not practice KMC. Thus, during KMC programmes, it may be beneficial to provide specific information to mothers on how to interact and take care of their preterm infants. This could decrease maternal insecurities and therefore increase positive perceptions (Pascoe et al., 2016), abilities and wellbeing.

## Implications and recommendations

The implications and recommendations derived from our scoping review facilitate the design and delivery of family-centred early intervention services that empower the mothers of preterm infants by making them active participants in the intervention process. The results of this scoping review indicate that the mothers of preterm infants mostly perceive the NICU and other hospital settings as a rule-bound and strict medical context where they become passive onlookers of their infants’ care. These perceptions worsen mothers’ existing negative emotions such as anxiety, insecurity and self-doubt. It seems that the guiding principle of family-centred early intervention (American Speech-Language-Hearing Association [ASHA], 2008) is not optimally implemented in this highly medical and technology-dominant environment. With regard to future research, it is thus recommended that in-depth primary research be conducted with perceptions of the mothers of preterm infants’ being the main source of data. A more specific recommendation would be to have focus groups or individual interviews with this vulnerable population to determine their information and support needs as well as the preferred method and agent of transfer of information. This specific research may guide the design and delivery of neonatal intervention programmes (McInroy & Kritzinger, 2005) that address the unique needs of this vulnerable population.

With regard to clinical practice, the following practical strategies are recommended to reduce negative maternal perceptions and overcome the various barriers that hospitalisation poses to natural interaction between the mother and infant dyad:

Firstly, it is advised that health professionals establish open and safe communication pathways with the mothers of preterm infants. This may empower mothers to actively seek the information and support they require. Secondly, health professionals could provide more information on general caregiving activities such as bathing or dressing of a preterm infant alongside more complex medical information. This would reduce maternal insecurities regarding the performance of activities of daily living. Health professionals could also encourage mothers’ participation during these activities to prevent their feelings of passive onlookers during their infants’ care and to build their confidence. Thirdly, it is recommended that health professionals encourage maternal participation and engagement during the feeding period. This recommendation is valid for all feeding methods, and is of special importance when infants are not able to breastfeed. The last recommendation is that special care must be taken to ensure that sufficient opportunities exist for interaction and bonding between the mother and infant dyad. The implementation of structured KMC programmes could facilitate mother–infant bonding and attachment, and aid in reducing negative maternal perceptions relating to their infant.

## Conclusion

This scoping review aimed to map and describe the time, setting, research methodology and results of the available literature in the field of early communication development and intervention in the preterm population, specifically maternal perceptions thereof. Firstly, it became evident that the defined research field is limited and does not provide sufficient insight into the maternal perceptions of early communication development and intervention. Secondly, it became clear that mothers of preterm infants experience various difficult emotions and realities whilst being hospitalised and taking care of their infants. The ecology of the NICU and other hospital settings is furthermore perceived as a barrier to natural interaction between the mother and infant dyad. It is thus recommended that in-depth research is conducted with this vulnerable population to determine their unique information and support needs as well as the preferred method and agent of transfer. Practical suggestions for clinical practice that aim to reduce negative maternal perceptions and overcome the perceived barriers that hospitalisation poses are further advised. This scoping review therefore strives to facilitate the design and delivery of family-centred early intervention services that empower the mothers of preterm infants by making them active participants in the intervention process.

## Appendix 1

**TABLE 1-A1:** References of 12 studies included in scoping review.

Number	Articles included in scoping review
1.	Crisp (2006)
2.	Feldman et al. (2002)
3.	Flacking et al. (2006)
4.	Green and Phipps (2015)
5.	Kritzinger and Louw (2003)
6.	Leonard and Mayers (2008)
7.	McInroy and Kritzinger (2005)
8.	Pascoe et al. (2016)
9.	Tanner (2012)
10.	Nicolaou et al. (2009)
11.	Swift and Scholten (2009)
12.	Kritzinger and Van Rooyen (2014)

Note: Please see the full reference list of the article, Van Schalkwyk, E., Gay, S., Miller, J., Matthee, E., & Gerber, B. (2020). Mothers with preterm infants’ perceptions of early communication development: A scoping review. *South African Journal of Communication Disorders, 67*(1), a640. https://doi.org/10.4102/sajcd.v67i1.640, for more information.

## Appendix 2

**TABLE 1-A2:** The format used to chart 12 articles.

Themes	Articles
Author(s); year of publication:	
Type of study:	
Describe the study population:	
Identify the study settings:	
Identify the research question:	
Identify the aim of the study:	
Define the type intervention used in the study: Comparisons identified in intervention methodology Participants Procedures used for data collection and analysis Materials/instruments used	
Relevant results:	
Limitations of study:	
Recommendations and implications:	
